# Solid-electrolyte interphase nucleation and growth on carbonaceous negative electrodes for Li-ion batteries visualized with *in situ* atomic force microscopy

**DOI:** 10.1038/s41598-020-65552-6

**Published:** 2020-05-22

**Authors:** Sergey Yu. Luchkin, Svetlana A. Lipovskikh, Natalia S. Katorova, Aleksandra A. Savina, Artem M. Abakumov, Keith J. Stevenson

**Affiliations:** Center for Energy Science and Technology, Skolkovo Institute of Science and Technology, Moscow, Russia

**Keywords:** Batteries, Batteries, Atomic force microscopy, Scanning electron microscopy

## Abstract

Li-ion battery performance and life cycle strongly depend on a passivation layer called solid-electrolyte interphase (SEI). Its structure and composition are studied in great details, while its formation process remains elusive due to difficulty of *in situ* measurements of battery electrodes. Here we provide a facile methodology for *in situ* atomic force microscopy (AFM) measurements of SEI formation on cross-sectioned composite battery electrodes allowing for direct observations of SEI formation on various types of carbonaceous negative electrode materials for Li-ion batteries. Using this approach, we observed SEI nucleation and growth on highly oriented pyrolytic graphite (HOPG), MesoCarbon MicroBeads (MCMB) graphite, and non-graphitizable amorphous carbon (hard carbon). Besides the details of the formation mechanism, the electrical and mechanical properties of the SEI layers were assessed. The comparative observations revealed that the electrode potentials for SEI formation differ depending on the nature of the electrode material, whereas the adhesion of SEI to the electrode surface clearly correlates with the surface roughness of the electrode. Finally, the same approach applied to a positive LiNi_1/3_Mn_1/3_Co_1/3_O_2_ electrode did not reveal any signature of cathodic SEI thus demonstrating fundamental differences in the stabilization mechanisms of the negative and positive electrodes in Li-ion batteries.

## Introduction

Li-ion battery (LIB) performance, life cycle, and safety strongly depend on interfacial processes in general and on solid-electrolyte interphase (SEI) in particular^1–3^. SEI is a product of electrolyte reduction on the negative electrode (anode) surface, which shifts the electrode potential into the electrolyte stability window^4^ thus preventing further electrolyte decomposition and stabilizing the electrode/electrolyte interface. The optimal SEI should possess negligible electrical conductivity and electrolyte permeability, high Li diffusivity, must accommodate mechanical expansion and contraction stress without cracking and delamination, be insoluble in the electrolyte in a working range of potentials and temperatures. Poorly formed SEI leads to rapid fade of electrochemical capacity. Even optimal SEI is suffering from continuous growth during cycling, which consumes Li and increases cell impedance resulting in gradual fade of capacity and power density^5^. Above all, in commercial production the initial SEI formation process is expensive and time consuming^6^. Therefore, thorough understanding of SEI formation mechanism is essential for finding optimal SEI formation protocols, increasing battery performance and lowering cost.

SEI structure and chemical composition are studied in great details^1,2,7^. In contrast, its formation mechanism remains elusive due to difficulty of *in situ* measurements of multicomponent battery electrodes^8^. The SEI formation is often observed by *in situ* Atomic Force Microscopy (AFM). Until now most of *in situ* AFM measurements were conducted on a basal plane of Highly Oriented Pyrolytic Graphite (HOPG)^9–15^. However, Li intercalates through the edge plane, SEI on which must determine battery properties to higher extent than the basal plane SEI. It is known that SEI composition^2,16,17^ and a first cycle capacity loss^18–20^ depend on type and quality of carbon which determines the edge to basal plane ratio. Earlier salt reduction at the edge plane favors larger content of inorganic components in a SEI^1,16,21,22^. Preferential solvent reduction on the basal plane at lower potential vs. Li^+^/Li determines larger organic content in the SEI^1^. Besides, the SEI growth and structure depend on binder material^23,24^. To the best of our knowledge, only two *in situ* AFM measurement of SEI formation were reported on a composite electrode comprised of graphite powder mixed with polyvinylidene difluoride (PVDF) binder and a conductive additive: (1) in 1997^25^ authors reported difficulties of such measurements, and (2) in 2017^26^ the imaging quality was not enough to observe SEI formation due to rough surface and AFM tip contamination.

Not only anodic SEI but also cathodic electrolyte interface (CEI) is under ongoing investigation especially relevant for emerging high voltage materials, where 4.7 V vs. Li^+^/Li oxidation potential of common organic electrolytes may be surpassed. Different mechanisms underlying the first cycle irreversibility in layered oxides have been discussed, including formation of CEI^27–34^, side reactions^35^, and structural transformations^36–38^. The experimental results are still sparse due to absence of model samples such as HOPG and due to difficulty of *in situ* AFM measurements of powder samples.

Here we provide a facile methodology for *in situ* atomic force microscopy (AFM) measurements of SEI formation on cross-sectioned composite battery electrodes allowing for direct observations of SEI formation on various types of carbonaceous negative electrode materials for Li-ion batteries. Using this approach, we observed SEI nucleation and growth on highly oriented pyrolytic graphite (HOPG), MesoCarbon MicroBeads (MCMB) graphite, and non-graphitizable amorphous carbon (hard carbon). Besides the details of the formation mechanism, the electrochemical and mechanical properties of the SEI layers were assessed. The comparative observations revealed that the electrode potentials for SEI formation differ depending on the nature of the electrode material, whereas the adhesion of SEI to the electrode surface clearly correlates with the surface roughness of the electrode. Finally, the same approach applied to a positive LiNi_1/3_Mn_1/3_Co_1/3_O_2_ electrode did not reveal any signature of cathodic SEI thus demonstrating fundamental differences in the stabilization mechanisms of the negative and positive electrodes in Li-ion batteries.

## Electrochemical cell

**Figure 1:**
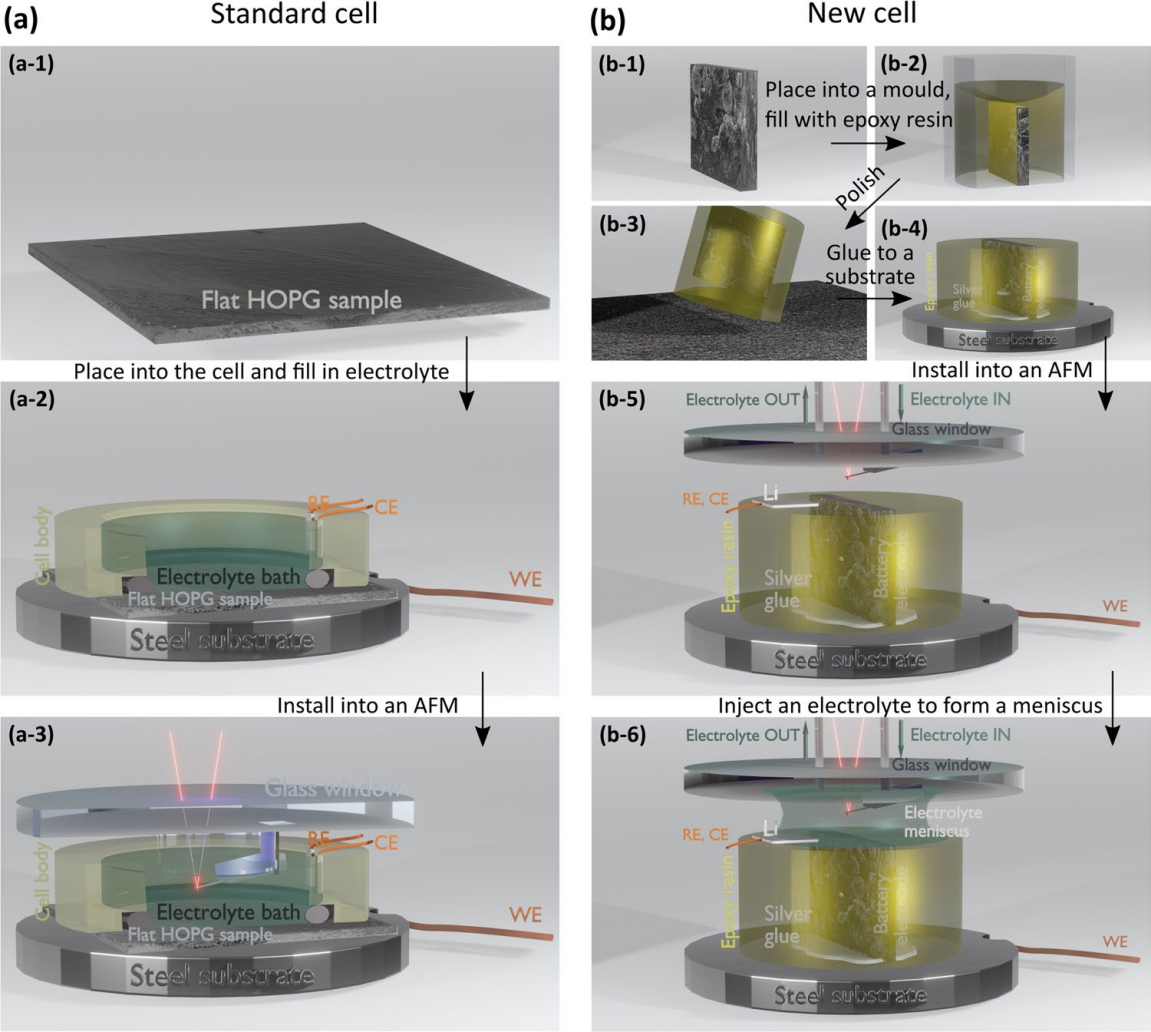
Schematic illustration of a standard (**a**) and the new (**b**) AFM electrochemical cells. In the standard cell the flat sample (a-1) is fixed and sealed at the bottom of the cell (a-2). The cell body is filled with the electrolyte and the cantilever is immersed in the electrolyte bath (a-3) for scanning. In the new cell the sample (b-1) is embedded into epoxy resin (b-2), polished/cross-sectioned (b-3), connected to the substrate by a conductive silver or carbon paint (b-4), and installed into an AFM. The cantilever is positioned above the sample (b-5), and the electrolyte is injected through the tubing to form the meniscus between the sample and the cantilever holder (b-6). The images were created in Blender 2.8 software (https://www.blender.org/).

In order to measure SEI formation on cross-sections of composite battery electrodes *in situ* in AFM, we designed a new electrochemical cell on the basis of a liquid perfusion cell capable to measure bulky samples. Figure 1 schematically illustrates a standard AFM electrochemical cell and the new one. In the standard cell (Fig. 1(a)) a flat sample is clamped at the bottom of the cell body, sealed by an o-ring, and connected as a working electrode (WE) to an external potentiostat/galavanostat. The cell body with counter (CE) and reference (RE) electrodes is filled with an electrolyte and a cantilever is immersed into the electrolyte bath for scanning. The cell body is typically made of Polyether ether ketone (PEEK) and Polytetrafluoroethylene (PTFE), which possess high chemical resistance to a wide range of chemical compounds. This cell configuration allows measurements only on flat samples such as HOPG.

In the new cell (Fig. 1(b)) measurements are performed in the electrolyte meniscus formed between the sample and the cantilever holder. Thus the sample may be bulky and does not require sealing by an o-ring, which allowed us to use cross-sections of composite battery electrodes embedded in epoxy resin. The epoxy resin fixes the composite electrode sample, and its polished surface additionally serves as a support for the electrolyte meniscus and the reference and counter electrodes. The meniscus is formed by injecting the electrolyte through the tubing fixed from the top in the window of the cantilever holder.

## Influence of sample preparation on the surface state

**Figure 2:**
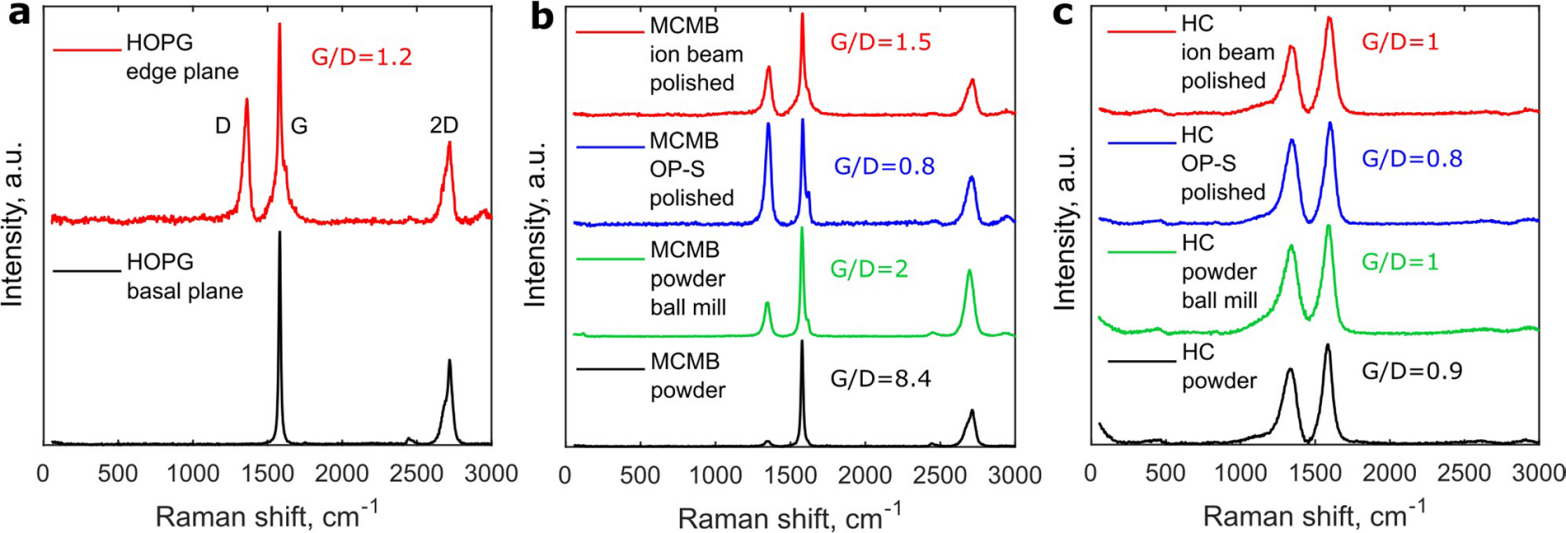
Raman spectroscopy of HOPG (**a**), MCMB (**b**), and HC (**c**) samples after different surface treatment. Ratio of area below D and G bands is indicated. For the HC ball milling, mechanical, and ion beam polishing did not significantly change surface state and the corresponding Raman spectrum. For the MCMB ball milling, mechanical, and ion beam polishing resulted in increased D-band intensity and corresponding increase of the amount of non-basal planes of graphite.

After preparation of the cross-sections, described in details in the Methods section, we analyzed to what extent the polished surface is equal to the untreated surface of the original powder. Figure 2 illustrates normalized Raman spectrum for HOPG, MCMB, and HC. The fresh HOPG basal plane gave intense G-band and no D-band. The Ar ion beam polished edge surface of the HOPG gave additional D-band with the G- to D-band area ratio equal 1.2. D and G bands from HC were similar from the pristine powder, ball-milled powder, the mechanically polished surface after the final polishing with the active oxide polishing suspension (OP-S, Struers), and the ion beam polished surface. On the pristine MCMB powder the D- to G-band ratio was 8.4. After the ball milling its ratio dropped to 2. After the mechanical polishing the D- to G-band ratio dropped to 0.8 and after the ion beam polishing slightly increased to 1.5. Both the HOPG and the MCMB possessed the 2D bands, while the HC did not.

The results show that the HC surface after the ball milling, mechanical, and ion beam final polishing closely resemble the pristine powder surfaces. On the contrary, the ratio of basal to non-basal planes on the MCMB surface (correlates with the G- to D-band ratio^19^) strongly drops already after the ball milling, which is an inherent step of the battery electrode production. Additional mechanical polishing further slightly reduces this ratio, and the following ion beam polished slightly increases it, making similar to the ion-beam polished HOPG edge plane. Overall, on the MCMB the polished surface resemble the ball milled one which is utilized in a commercial battery. Taking into account that the SEI composition on the HOPG edge plane, hard carbon, and soft carbon is similar with somewhat smaller content of salt reduction products in the soft carbon SEI^2^, the SEI from the cross-sections must be more representative for real battery SEI than the SEI from the basal plane HOPG.

Due to higher roughness of the ion beam polished samples for further study we used the OP-S polished samples.

## *In situ* SEI formation on HOPG, MesoCarbon MicroBeads (MCMB) graphite, and hard carbon (HC)

**Figure 3:**
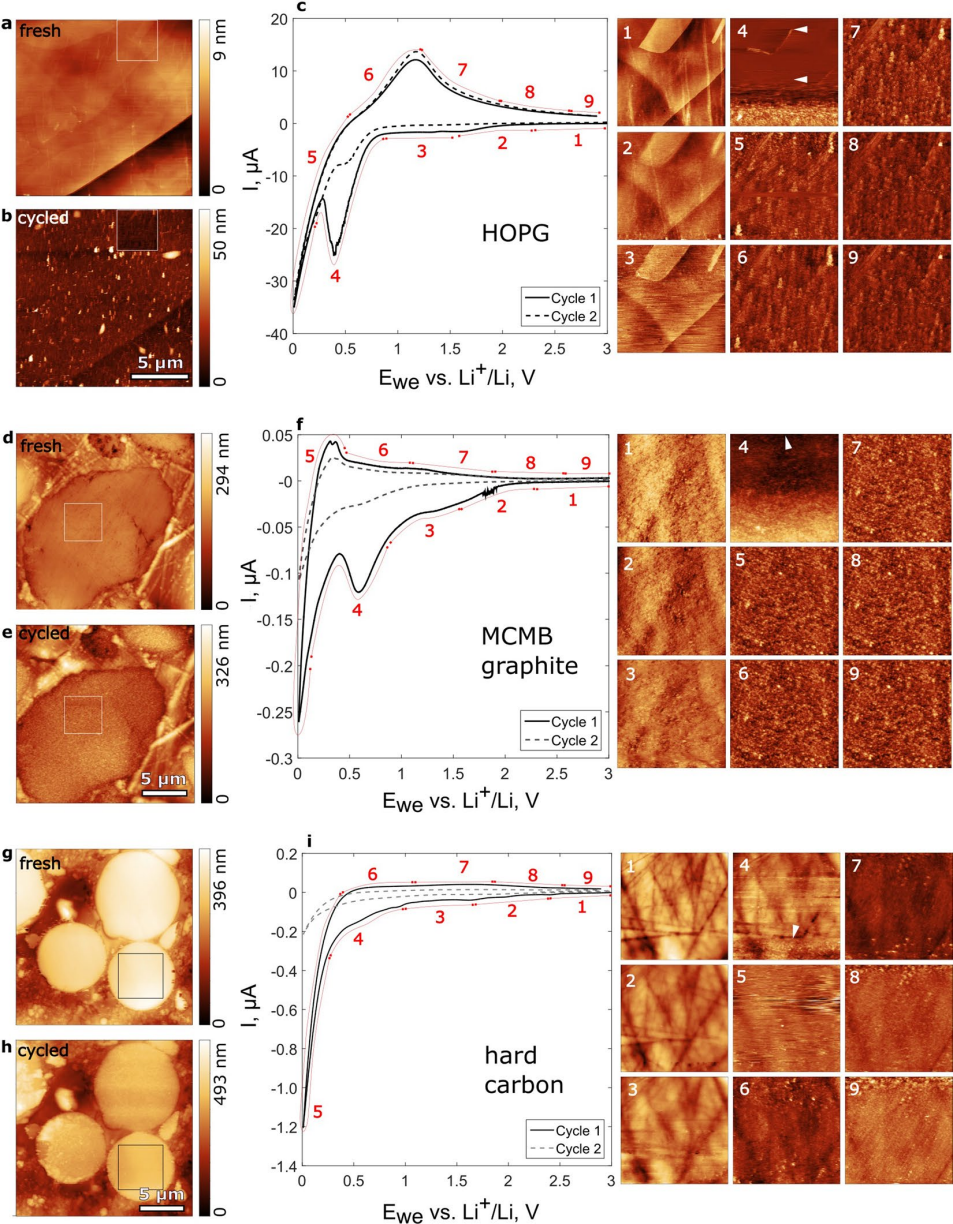
CV curves and corresponding AFM topography images recorded on HOPG (**a**–**c**), MCMB graphite (**d**–**f**), and hard carbon (**g**–**i**) in 1 M LiPF_6_ in EC/DMC = 50/50 (v/v) electrolyte solution at 2 mV/s cycling rate vs Li^+^/Li. HOPG before (**a**) and after (**b**) cycling. MCMB graphite before (**d**) and after (**e**) cycling. Hard carbon before (**g**) and after (**h**) cycling. 4 × 4 µm squares in (**a,b**), (**d,e**), and (**g**,**h**) depict *in situ* scan areas shown in (**c**), (**f**), and (**i**) correspondingly as 1–9. White arrows indicate SEI nucleation.

Figure 3 shows comparative cyclic voltammetry (CV) curves and corresponding *in situ* AFM images of the HOPG, MCMB, and HC surface before, during, and after cycling. The HOPG was used as a reference sample. Its freshly cleaved surface shown in Fig. 3(a) is the graphite basal plane with small fraction of edge sites along step edges. The MCMB and HC samples shown in Fig. 3 (d,g), respectively, are cross sections of composite electrodes made of powder mixed with polyvinylidene difluoride (PVDF) binder and Super P carbon black embedded in epoxy resin. Being rougher than the HOPG, such cross sections are sufficiently flat for AFM imaging.

**Figure 4:**
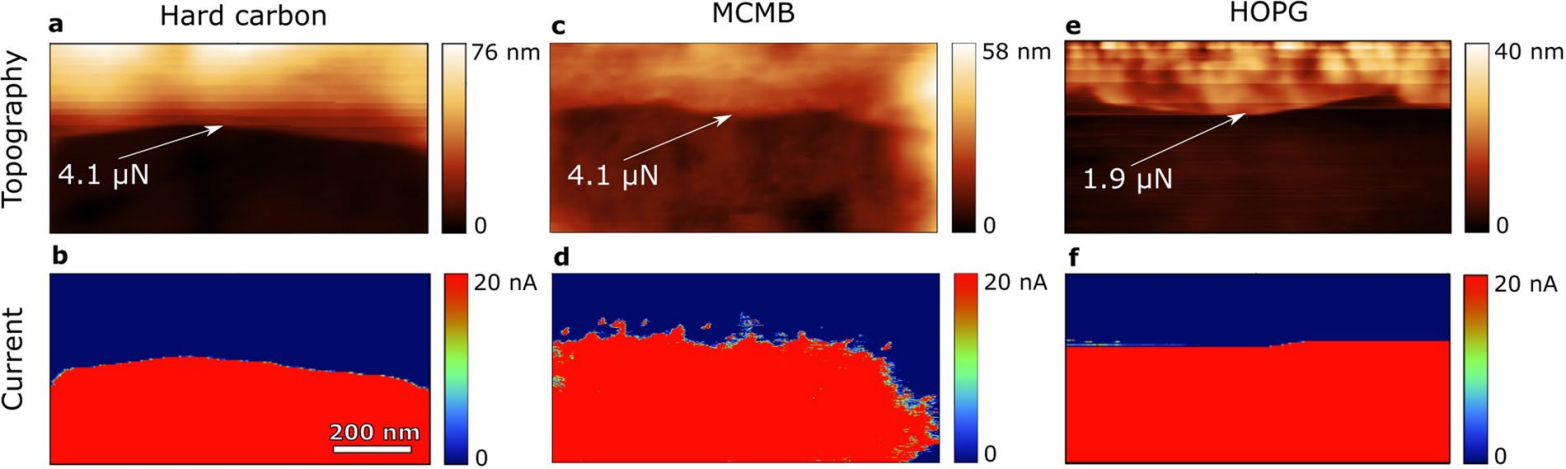
Topography and current maps of hard carbon (**a,b**), MCMB graphite (**c,d**), and HOPG (**e,f**) obtained during scratching the SEI layer with a diamond coated conductive cantilever in the conductive AFM mode. Numbers with arrows indicate force at which and place where the SEI was scraped off, which corresponds to the current onset.

During the first CV cycle on HOPG, SEI was detected along step edges at 0.8 V and on the basal plane at 0.5 V (Fig. 3(c-4)). This process is associated with the first current peak at 0.8–0.3 V. At about 0.4 V the SEI merged into a uniform layer and its topography remained stable during further cycling. Figure 3(b) shows the formed SEI after the first CV. Apart from the SEI we observed blisters with lateral size up to 2 µm and height up to 35 nm. During the second CV (dashed line in Fig. 3(c)) the SEI morphology did not change and the 0.8–0.3 V current peak was 3 times smaller.

The observed earlier SEI nucleation at the edge carbon sites is in agreement with previous studies showing that the edge plane possess higher electrocatalytic activity than the basal plane^21,22^, which facilitates earlier salt reduction and favors larger content of inorganic components such as LiF, Li_2_O, and Li_2_CO_3_ in a SEI in LiPF_6_-containing electrolyte^1,16^ and higher thickness^2^. Preferential solvent reduction on the basal plane at lower potential vs. Li^+^/Li determines larger organic content in the SEI.

Likewise, blistering is a typical phenomenon in ethylene carbonate (EC)-based electrolytes^39–41^. It is caused by cointercalation of electrolyte molecules and trace water into graphite structure and consequent gas evolution on the cathodic scan^42^. Indeed when we used water contaminated electrolyte and observed a water reduction current peak at 1.3 V^13,43–45^, blistering was more intense and particularly active on the cathodic cycle (Supplementary Fig. 1).

On the MCMB sample the SEI nucleation was detected at about 0.9 V of the first CV cycle (Fig. 3(f-4)). At 0.6 V the SEI formed a uniform layer, which stabilized at about 0.3 V. The process is associated with the current peak at 1.0–0.4 V. The formed SEI morphology remained stable during further cycling. During the second CV (dashed line in Fig. 3(f)) the SEI morphology did not change and the current peak was almost 5 times smaller.

Because the MCMB graphite particle is a mixture of nanosized regions with different orientations, the edge to basal sites ratio on the surface is much larger than on HOPG, which naturally explains the 0.9 V SEI nucleation potential – close to the step edge SEI nucleation on the HOPG. Consequently, we can expect more inorganic fraction in the SEI.

On the HC sample distinct but loosely bound surface deposit appeared at about 1 V. It was permanently scraped off by the cantilever until a complete SEI layer was formed at about 0.4 V (Fig. 3(i-4)). Its topography further remained unchanged. The process was associated with the gradual current increase on the stage 4 (Fig. 3(i)) without distinct anodic peak. A larger area scan after the first CV (Fig. 3(h)) revealed partial SEI delamination on the particles on the left hand side. Thickness of the delaminated SEI was about 70 nm. After the second CV the SEI morphology remained unchanged but more rough atop the delaminated regions (Supplementary Figure 2).

Similarly to the edge plane of graphite, disordered structure of hard carbon facilitates its electrocatalitic activity and results in SEI composition similar to one on the edge plane of HOPG and graphite^2^. However, we reproducibly observed SEI formation at about 0.4 V vs. Li^+^/Li. We suggest that due to flat surface of hard carbon (Rms = 1.4 nm) and weak adsorption, the inorganic products of salt reduction were removed from the surface by the AFM tip even in the gentle tapping mode, which was manifested as the loosely bound surface deposit in the 1.0–0.4 V range. The appearance of organic products of electrolyte reduction below 0.5 V must have enhanced adhesion and allowed SEI anchoring on the HC surface.

The samples with formed SEI were gently washed in dimethyl carbonate in order to remove remaining salt, and dried in the Ar filled glovebox. The SEI then was scratched by a stiff (98 N/m spring constant) diamond coated conductive cantilever with 100 nm tip radius in a conductive AFM mode. The moment of reaching carbon through the SEI was detected by onset of electric current at 0.5 V electric bias applied between the sample and the cantilever. Such approach allows excluding misinterpretation of the dense lower SEI layer with the electrode surface.

Figure 4 illustrates topography and current maps of the samples during scratching with the applied force increasing from top to bottom. The SEI was scraped off when the force reached 1.9 µN on HOPG and 4.1 µN on MCMB and on HC. This difference is naturally explained by the fact that the SEI formed on edge plane sites of MCMB and on disordered sites of HC is rich in inorganic salt reduction products, while the SEI on the HOPG basal plane is rich in polymer products^1,16^. Moreover, when the force exceeded the threshold value, the SEI readily peeled off from the HC (Fig. 4(a,b)) and from HOPG (Fig. 4(e,f)), showing smooth edge on the current maps, while on the MCMB graphite the edge was rough (Fig. 4(c,d)). It suggests that the SEI is better bound to the rough surface of MCMB than to smooth surface of the HC and the basal plane of HOPG.

## SEM characterization of SEI cross-sections

The washed samples with SEI were transferred in a sealed vial to another N_2_ filled glovebox with a physical vapor deposition chamber installed inside, where a 100 nm thick Al coating was deposited on the samples in order to protect the SEI from exposure to atmosphere during transition into the Helios PFIB G4 UXe dual beam system. During the deposition the samples’ temperature did not exceed 60 °C.

**Figure 5:**
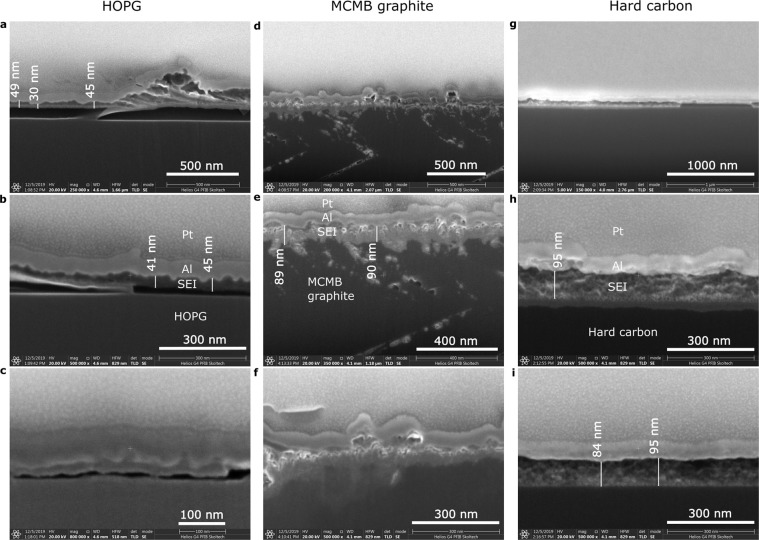
Scanning electron microscopy images of FIB cross sections of HOPG (**a–c**), MCMB graphite (**d–f**), and HC (**g–i**) samples with SEI. The SEI on HC and MCMB is about two times thicker than on HOPG. On the MCMB the SEI is additionally rooted into porosity.

Figure 5 illustrates cross-sections of the samples exposing host electrode material (HOPG, HC, and MCMB), SEI layer, protective Al coating, and Pt layer deposited prior to focused ion beam milling. On the HOPG we can clearly distinguish a blister (Fig. 5(a,b)) and delaminated SEI (Fig. 5(a–c)). The delamination was probably caused by vertical tension stress developed during blistering. The SEI is integral with thickness about 45 nm. On the HC the SEI in about 90 nm thick – twice thicker than on the HOPG basal plane – and possess smooth interface with HC. Partial SEI delamination is observed in Fig. 5(g). On the MCMB the SEI is about 90 nm thick – similar to the HC. However, the SEI/MCMB interface is drastically different: the SEI fills pits in porous graphite surface and thus is pinned to the surface. Such interface structure must reinforce the SEI/MCMB contact and reduce possibility of delamination. It is also consistent with the AFM scratching results – Fig. 4(d) – and correlates with surface roughness: root mean squared (Rms) roughness of the fresh *in situ* scanned regions of 4 × 4 µm^2^ of HOPG, HC, and MCMB are 0.1 nm, 1.4 nm, and 3.2 nm respectively. Thus, sufficiently rough surface of particles benefits stronger contact with SEI.

## *In situ* AFM investigation of NMC 111 surface during cycling

**Figure 6:**
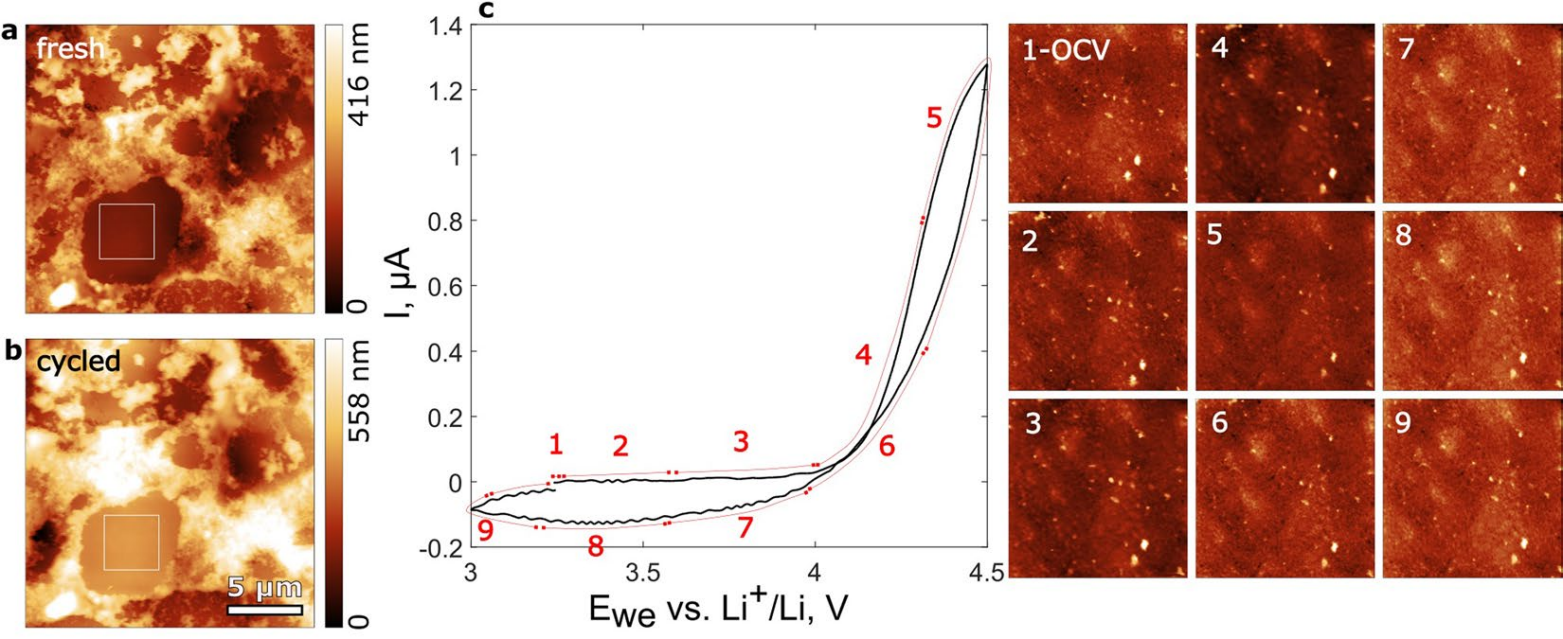
CV curve and corresponding AFM topography images recorded on LiCo_1/3_Ni_1/3_Mn_1/3_O_2_ in 1 M LiPF_6_ in EC/DMC = 50/50 (v/v) electrolyte solution at 2 mV/s cycling rate vs Li^+^/Li. LiCo_1/3_Ni_1/3_Mn_1/3_O_2_ before (**a**) and after (**b**) cycling. 4 × 4 µm squares in (**a**) and (**b**) depict *in situ* scan areas shown in (**c**).

Using our approach, we performed *in situ* electrochemical AFM measurements on a cross section of a composite cathode made of LiCo_1/3_Ni_1/3_Mn_1/3_O_2_ (NMC 111) powder mixed with PVDF binder and Super P carbon black. Figure 6 shows a cyclic voltammetry (CV) curve and corresponding AFM images of the NMC 111 surface before, during, and after cycling. We did not observe SEI formation or any deposit on the surface in the whole 3.0–4.5 V potential range. Instead, comparison of Fig. 6(a,b) revealed changes in morphology of secondary particles, associated with anisotropic lattice contraction caused by Li deintercalation from NMC 111 particles (more details in Supplementary Fig. 3). The CV curve demonstrates a partial current peak associated with Li deintercalation from NMC 111 and low and broad intercalation current, different from the macroscopic CV. The latter may be due to flat electrode geometry and size effect^46^.

## Conclusion

In this work we proposed a new methodology for *in situ* atomic force microscopy (AFM) measurements of SEI formation on cross-sectioned composite battery electrodes allowing for direct observations of SEI formation on various types of carbonaceous negative electrode materials for Li-ion batteries. Using this approach, we observed and compared SEI nucleation and growth on highly oriented pyrolytic graphite (HOPG), MesoCarbon MicroBeads (MCMB) graphite, and non-graphitizable amorphous carbon (HC). We found that under given experimental conditions SEI on the edge sites of HOPG nucleated at about 0.8 V and on the basal plane sites at about 0.5 V. On the MCMB graphite SEI appeared at 0.8–0.9 V and on HC at about 0.4 V with the preliminary weakly bound deposit at about 1 V. The SEI on both MCMB and HC were twice thicker – 90 nm vs 45 nm – and mechanically stronger – 4.1 µN vs 1.9 µN – than the SEI on HOPG. These findings are in good agreement with previous studies showing that SEI on the edge plane is rich in inorganic salt reduction products, while the SEI on the basal plane is rich in polymer products. Moreover, we found that smooth SEI/HOPG and SEI/HC interface is prone to delamination, while rough SEI/SG interface is less so due to SEI penetration into surface porosity. Finally, the same approach applied to a positive LiNi_1/3_Mn_1/3_Co_1/3_O_2_ electrode did not reveal any signature of cathodic SEI thus demonstrating fundamental differences in the stabilization mechanisms of the negative and positive electrodes in Li-ion batteries.

## Methods

### Materials synthesis

The layered cathode material LiNi_1/3_Co_1/3_Mn_1/3_O_2_ (NMC 111) was synthesized by calcination of the precursor prepared using a co-precipitation method. First, a 2 M aqueous solution of Mn^2+^, Ni^2+^, and Co^2+^ was prepared from NiSO_4_·6H_2_O (RusKhim), MnSO_4_·H_2_O (RusKhim), and CoSO_4_·7H_2_O (RusKhim) in a 1:1:1:stoichiometric ratio. The solution was pumped into a Batch reactor (20 L) under N_2_ atmosphere. At the same time, an alkali solution with 2 M Na_2_CO_3_ (RusKhim) and 0.3 M NH_4_OH was also dropped into the reactor. The pH value, temperature, and stirring speed were carefully controlled. Then, the co-precipitated particles were obtained after filtering, washing with deionized water, and drying at 90–110 °C in a vacuum oven. Finally, LiNi_1/3_Co_1/3_Mn_1/3_O_2_ was prepared by annealing the dried precursor with 6% excess of LiOH·H_2_O (RusKhim) at 500 °C for 5 h in air, and then at 850 °C for 12 h in air.

The hard carbon (HC) powder was prepared by a hydrothermal synthesis from D-glucose (Sigma Aldrich, >99.5%) followed by pyrolysis. 9 g of D-glucose was mixed with 0.1 g of pectin (Souzopttorg, ARA104) and dissolved in 25 ml of deionized water. The mixture was placed in a Teflon lined stainless steel autoclave reactor with addition of 2 ml polytetrafluoroethylene solution (PFTE: Sigma Aldrich, 60 wt. % dispersion in H_2_O) and the synthesis was performed at 180 °C for 8 hours. The obtained powder was centrifuged, washed in deionized water, dried in air, and finally annealed in a tubular furnace with Af flow at 1200 °C for 5 h.

MesoCarbon MicroBeads (MCMB) D-10 graphite powder was purchased from Gelon Lib Group.

### Materials characterization

The LiNi_1/3_Co_1/3_Mn_1/3_O_2_ powder was characterized by X-ray diffraction using a Huber G670 Guinier diffractometer (CoKα1 radiation (λ = 1.78892 Å), curved Ge(111) monochromator, image plate detector). To determine the lattice parameters, the Le Bail decomposition was carried out using the JANA2006 software. The XRD spectra is presented in Supplementary Fig. 4.

The hard carbon powder was characterized by scanning electron microscopy using Quattro S ESEM (FEI) and Raman spectroscopy using DXRxi Raman Imaging Microscope (Thermo Fisher Scientific). The results presented in Supplementary Fig. 5 illustrate a characteristic round particle of hard carbon and its Raman spectra with D-band (defect-induced) and G-band (crystalline graphite) peaks at ~1353 cm^−1^ and ~1585 cm^−1^ respectively.

Raman spectroscopy on the HOPG, pristine powders, and cross-sections was performed using DXRxi Raman Imaging Microscope (Thermo Fisher Scientific) using the 532 nm laser.

### Sample preparation

MCMB, HC, and NMC 111 powders were separately mixed with PVDF and super P carbon black conductive additive in 80:10:10 mass ratio in N-Methyl-2-pyrrolidone (NMP) solvent and homogenized in a ball mill (Spex 8000 M) for 20 minutes. The slurry was deposited onto a polyimide tape (Kapton) and dried in a vacuum oven at 50 °C overnight. Small pieces (up to 5 × 5 mm) of the dried composite electrodes were delaminated from the polyimide substrate by a tweezer and placed in a mold, which was made by cutting a piece of a silicone tubing with 10 mm internal diameter. The mold with the electrode was filled with a bisphenol A/F epoxy resin HT2 with hardener HT2 (R&G Faserverbundwerkstoffe GmbH, Germany), placed in a vacuum oven for vacuum infusion in order to fill porosity in the electrodes, and cured under ambient conditions according specification. Diameter of the mold was chosen considering size of a sample stage of the AFM.

After curing and hardening the samples were mechanically polished on a SiC sand paper (25 µm and 10 µm particle size), diamond suspension (3 µm and 0.25 µm particle size), and OP-S silica suspension (Struers, 40 nm particle size) consequently. The last step provides high quality surface comparable with one obtained after chemical etching^47^. (Additional cross-sectional samples for Raman imaging were further polished by an Ar ion beam (Leica EM RES102) with a 10 min cleaning step at 10° and a 15 min polishing step at 4°.) The polished samples were carefully washed in deionized water, dried in a stream of N_2_, and fixed from the bottom side on a steel substrate with conductive silver paint. The perimeter of the samples on the substrate was additionally sealed with bisphenol A/F or TorrSeal epoxy resin in order to prevent accidental electrolyte leak to the contact and dissolution of the silver paint. After transfer to the glove box, the samples’ surface was additionally washed with DMC. The HOPG ZYA sample was fixed on a steel substrate in the same way. Fresh surface was exposed before measurements by peeling off the top layer by a scotch tape. The prepared samples are shown in Supplementary Fig. 6.

Bisphenol A/F epoxy resin was chosen considering its electrochemical stability in the 1 M LiPF_6_ in EC/DMC = 50/50 (v/v) electrolyte solution. Bisphenol A is used for sealing microelectrodes for electrochemical applications^48,49^. Additional Bisphenol F reduces viscosity of the epoxy resin. The epoxy resin was thoroughly tested before measurements. First, the embedded samples were stored for 2 weeks in the closed vial filled with the electrolyte solution without visible changes. Second, 4 coin cells with the NMC 111 cathode and Li anode were assembled: 2 with and 2 without pieces of epoxy inside (weight of the epoxy pieces (≈3 mg) was comparable to the weight of the active NMC 111 powder (4.1 mg)). The cells were cycled between 2.8 V and 4.2 V at 0.3C-rate for 30 cycles. Resulting potential profiles and capacity were similar for cells with and without epoxy (Supplementary Fig. 7).

### *In situ* AFM measurements

*In situ* AFM measurements were performed in a tapping mode using Cypher ES microscope (Asylum Research, Oxford Instruments) installed inside an Ar filled glove box (MBraun) with O_2_ < 0.1 ppm and H_2_O < 0.1 ppm. A Si cantilever with 140 kHz resonance frequency and 0.6 N/m spring constant was mounted in a liquid perfusion cantilever holder and installed in an environmental sample cell. Before measurements the cantilever was washed with acetone and deionized water. An external potentiostat/galavanostat (BioLogic SP 150) was connected to the microscope. The samples were connected as working electrodes and a Li foil was connected as a reference and a counter electrode in a two electrode configuration. The cantilever was brought to a distance of 100 µm from the sample surface. Commercial battery grade electrolyte solution (1 M LiPF_6_ in EC/DMC = 50/50 (v/v)) (Sigma Aldrich) was injected between the sample and the cantilever holder by a syringe via a polyethylene tubing until it formed a meniscus between the sample and the fused silica window of the cantilever holder. After that the cantilever was lander on the sample surface and the measurements were performed with 512 × 512 pixels resolution. The detailed setup is illustrated in Supplementary Fig. 8.

AFM images were processed using Gwyddion software.

### Scanning electron microscopy

Scanning electron microscopy (SEM) images were obtained using Thermo Scientific Helios PFIB G4 UXe dual beam system in secondary electrons (SE) mode. The accelerating voltage was 5 kV and 20 kV, electron beam current was 0.1 nA. A sample tilt of cross-section images was 52°.

Sample cross-sections were obtained by focused ion beam (FIB) under high vacuum. First, a Pt protective layer was deposited at 12 kV and 1 nA on top of the Al cover layer. Then, cross-sections were milled at 30 kV and 4 nA. Finally, the cross-sections were cleaned at 30 kV and 0.3 nA in order to obtain a smooth surface.

## Supplementary information

Supplementary Information.

## Data Availability

The data are available from the corresponding author upon reasonable request.

## References

[1] An SJ (2016). The state of understanding of the lithium-ion-battery graphite solid electrolyte interphase (SEI) and its relationship to formation cycling. Carbon.

[2] Peled E, Menkin S (2017). Review—SEI: Past, Present and Future. J. Electrochem. Soc..

[3] Daniel, C. & Besenhard, J. O. *Handbook of battery materials*. (Wiley-VCH Verlag, 2011).

[4] Goodenough JB, Manthiram A, Wnetrzewski B (1993). Electrodes for lithium batteries. J. Power Sources.

[5] Pinson MB, Bazant MZ (2012). Theory of SEI Formation in Rechargeable Batteries: Capacity Fade, Accelerated Aging and Lifetime Prediction. J. Electrochem. Soc..

[6] Wood DL, Li J, Daniel C (2015). Prospects for reducing the processing cost of lithium ion batteries. J. Power Sources.

[7] Wang, A., Kadam, S., Li, H., Shi, S. & Qi, Y. Review on modeling of the anode solid electrolyte interphase (SEI) for lithium-ion batteries. *npj Computational Materials* **4** (2018).

[8] Tripathi AM, Su WN, Hwang BJ (2018). *In situ* analytical techniques for battery interface analysis. Chemical Society Reviews.

[9] Alliata D, Kötz R, Novák P, Siegenthaler H (2000). Electrochemical SPM investigation of the solid electrolyte interphase film formed on HOPG electrodes. Electrochem. commun..

[10] Shen C, Wang S, Jin Y, Han WQ (2015). *In Situ* AFM Imaging of Solid Electrolyte Interfaces on HOPG with Ethylene Carbonate and Fluoroethylene Carbonate-Based Electrolytes. ACS Appl. Mater. Interfaces.

[11] Yang G (2017). Improving the cyclability performance of lithium-ion batteries by introducing lithium difluorophosphate (LiPO2F2) additive. RSC Adv..

[12] Liu T (2019). *In situ* quantification of interphasial chemistry in Li-ion battery. Nat. Nanotechnol..

[13] Cresce AV, Russell SM, Baker DR, Gaskell KJ, Xu K (2014). *In situ* and quantitative characterization of solid electrolyte interphases. Nano Lett..

[14] Seidl L, Martens S, Ma J, Stimming U, Schneider O (2016). *In situ* scanning tunneling microscopy studies of the SEI formation on graphite electrodes for Li+ -ion batteries. Nanoscale.

[15] Kempaiah R, Vasudevamurthy G, Subramanian A (2019). Scanning probe microscopy based characterization of battery materials, interfaces, and processes. Nano Energy.

[16] Bar-Tow D, Peled E, Burstein L (1999). Study of highly oriented pyrolytic graphite as a model for the graphite anode in Li-ion batteries. J. Electrochem. Soc..

[17] Weber I, Schnaidt J, Wang B, Diemant T, Behm RJ (2019). Model Studies on the Solid Electrolyte Interphase Formation on Graphite Electrodes in Ethylene Carbonate and Dimethyl Carbonate: Highly Oriented Pyrolytic Graphite. ChemElectroChem.

[18] Winter M, Novák P, Monnier A (1998). Graphites for lithium-ion cells: The correlation of the first-cycle charge loss with the brunauer-emmett-teller surface area. J. Electrochem. Soc..

[19] Placke T (2012). Influence of graphite surface modifications on the ratio of basal plane to ‘non-basal plane’ surface area and on the anode performance in lithium ion batteries. J. Power Sources.

[20] Olivier JP, Winter M (2001). Determination of the absolute and relative extents of basal plane surface area and ‘non-basal plane surface’ area of graphites and their impact on anode performance in lithium ion batteries. in Journal of Power Sources.

[21] Yuan, W. *et al*. The edge- and basal-plane-specific electrochemistry of a single-layer graphene sheet. *Sci. Rep.* **3** (2013). 10.1038/srep02248 PMC3727060 23896697

[22] Velický M, Toth PS, Woods CR, Novoselov KS, Dryfe RAW (2019). Electrochemistry of the Basal Plane versus Edge Plane of Graphite Revisited. J. Phys. Chem. C.

[23] Young, B. T. *et al*. Role of binders in solid electrolyte interphase formation in lithium ion batteries studied with hard X-ray photoelectron spectroscopy. **34**, 97–106 (2019).

[24] Fransson L, Eriksson T, Edström K, Gustafsson T, Thomas J (2001). Influence of carbon black and binder on Li-ion batteries. J. Power Sources.

[25] Hirasawa, K. A., Sato, T., Asahina, H., Yamaguchi, S. & Mori, S. *In situ* electrochemical atomic force microscope study on graphite electrodes. *J. Electrochem. Soc*. **144** (1997).

[26] Shen C (2018). Direct Observation of the Growth of Lithium Dendrites on Graphite Anodes by Operando EC-AFM. Small. Methods.

[27] Lu W, Zhang J, Xu J, Wu X, Chen L (2017). *In Situ* Visualized Cathode Electrolyte Interphase on LiCoO2 in High Voltage Cycling. ACS Appl. Mater. Interfaces.

[28] Liu RR (2014). Facet dependent SEI formation on the LiNi0.5Mn1.5O4 cathode identified by *in situ* single particle atomic force microscopy. Chem. Commun..

[29] Zhang, S. S., Xu, K. & Jow, T. R. Formation of solid electrolyte interface in lithium nickel mixed oxide electrodes during the first cycling. *Electrochem. Solid-State Lett*. **5** (2002).

[30] Edström K, Gustafsson T, Thomas JO (2004). The cathode–electrolyte interface in the Li-ion battery. Electrochim. Acta.

[31] Simmen F (2011). Surface layer formation on Li1+xMn2O4−δ thin film electrodes during electrochemical cycling. Electrochim. Acta.

[32] Schulz N, Hausbrand R, Dimesso L, Jaegermann W (2018). XPS-surface analysis of SEI layers on Li-ion cathodes: Part I. Investigation of initial surface chemistry. J. Electrochem. Soc..

[33] Schulz N, Hausbrand R, Wittich C, Dimesso L, Jaegermann W (2018). XPS-surface analysis of SEI layers on Li-ion cathodes: Part II. SEI-composition and formation inside composite electrodes. J. Electrochem. Soc..

[34] Würsig, A., Buqa, H., Holzapfel, M., Krumeich, F. & Novák, P. Film formation at positive electrodes in lithium-ion batteries. *Electrochem. Solid-State Lett*. **8** (2005).

[35] Choi, J. & Manthiram, A. Investigation of the irreversible capacity loss in the layered LiNi 1/3Mn1/3Co1/3O2 cathodes. *Electrochem. Solid-State Lett*. **8** (2005).

[36] Arai H, Okada S, Sakurai Y, Yamaki JI (1997). Reversibility of LiNiO2 cathode. Solid State Ionics.

[37] Delmas C (1997). On the behavior of the LixNiO2 system: An electrochemical and structural overview. J. Power Sources.

[38] Kang Y-J, Kang J, Chang KJ (2008). Electronic structure of graphene and doping effect on SiO 2. Phys. Rev. B.

[39] Daniel, C. & Besenhard, J. O. Handbook of Battery Materials: Second Edition. 10.1002/9783527637188 (Wiley-VCH Verlag GmbH & Co. KGaA, 2011).

[40] Inaba M, Siroma Z, Kawatate Y, Funabiki A, Ogumi Z (1997). Electrochemical scanning tunneling microscopy analysis of the surface reactions on graphite basal plane in ethylene carbonate-based solvents and propylene carbonate. J. Power Sources.

[41] Song HY, Jeong SK (2018). Investigating continuous co-intercalation of solvated lithium ions and graphite exfoliation in propylene carbonate-based electrolyte solutions. J. Power Sources.

[42] Goss CA, Brumfield JC, Irene EA, Murray RW (1993). Imaging the incipient electrochemical oxidation of highly oriented pyrolytic graphite. Anal. Chem..

[43] Aurbach D, Daroux M, Faguy P, Yeager E (1991). The electrochemistry of noble metal electrodes in aprotic organic solvents containing lithium salts. J. Electroanal. Chem..

[44] Tang, M., Miyazaki, K., Abe, T. & Newman, J. Effect of graphite orientation and lithium salt on electronic passivation of highly oriented pyrolytic graphite. *J. Electrochem. Soc*. **159** (2012).

[45] Jeong, S. K., Inaba, M., Abe, T. & Ogumi, Z. Surface Film Formation on Graphite Negative Electrode in Lithium-Ion Batteries: AFM Study in an Ethylene Carbonate-Based Solution. *J. Electrochem. Soc*. **148** (2001).

[46] Gonzalez J, Molina A, Martinez-Ortiz F, Lopez-Tenes M, Compton RG (2016). Analytical approach to the transient and steady-state Cyclic Voltammetry of non-reversible electrode processes. Defining the transition from macro to microelectrodes. Electrochim. Acta.

[47] Koll L, Tsipouridis P, Werner EA (2011). Preparation of metallic samples for electron backscatter diffraction and its influence on measured misorientation. J. Microsc..

[48] Wightman RM (1981). Microvoltammetric electrodes. Anal. Chem..

[49] Zestos AG, Nguyen MD, Poe BL, Jacobs CB, Venton BJ (2013). Epoxy insulated carbon fiber and carbon nanotube fiber microelectrodes. Sensors Actuators, B Chem..

