# Supplementary material for: Solid-electrolyte interphase nucleation and growth on carbonaceous negative electrodes for Li-ion batteries visualized with in situ atomic force microscopy
Source: Sci Rep. 2020 May 22;10:8550. doi: 10.1038/s41598-020-65552-6 (PMC7244741; doi:10.1038/s41598-020-65552-6)
Supplement: Supplementary file 1 — Supplementary Information. [file 41598_2020_65552_MOESM1_ESM.pdf]

Supplementary Materials for

Solid-electrolyte interphase nucleation and growth on carbonaceous negative electrodes for Li-ion batteries visualized with *in situ* atomic force microscopy

Sergey Yu. Luchkin[1]*, Svetlana A. Lipovskikh[1], Natalia S. Katorova[1], Aleksandra A. Savina[1], Artem M. Abakumov[1], Keith J. Stevenson[1]

[1] Center for Energy Science and Technology, Skolkovo Institute of Science and Technology, Moscow, Russia

Contents:

Supplementary figures 1 to 8.

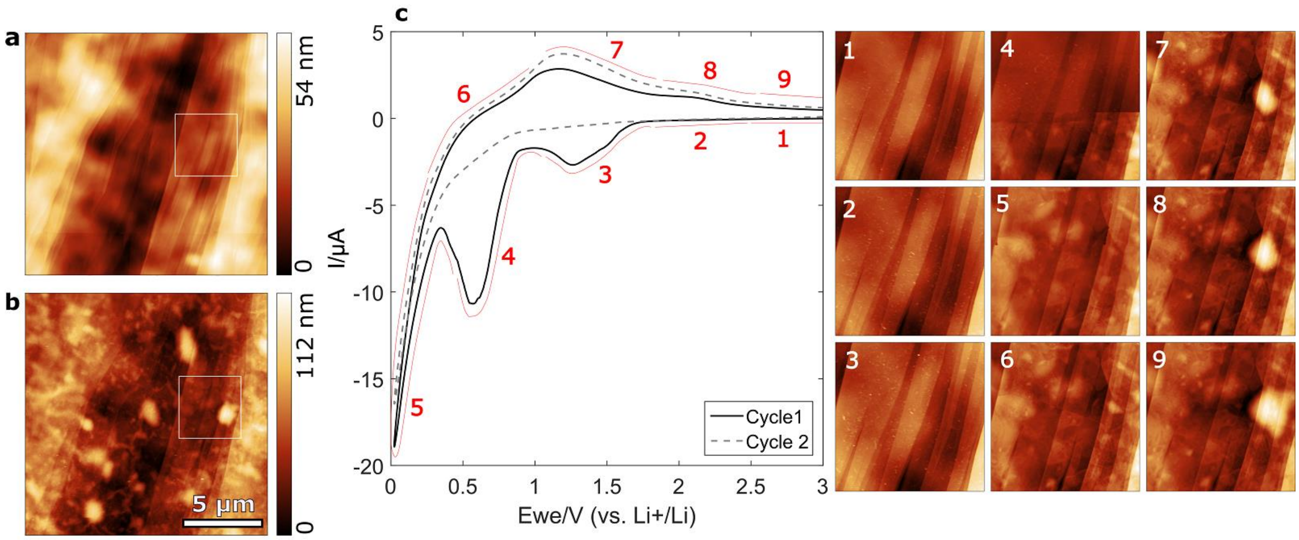

Supplementary figure 1. CV curve and corresponding AFM topography images recorded on HOPG at 2 mV/s cycling rate vs Li+/Li. 1.3 V current peak from water reduction. Active blister growth during the cathodic scan.

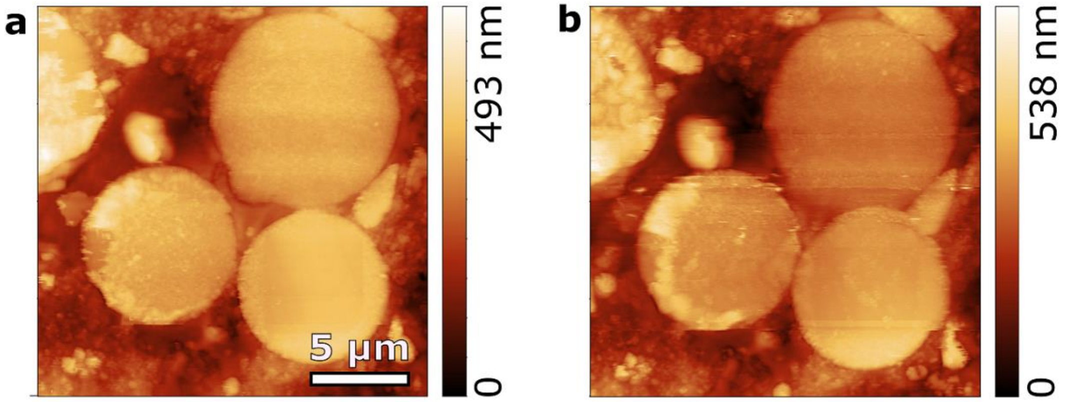

Supplementary figure 2. Surface topography of the hard carbon sample after the first (a) and the second (b) CV.

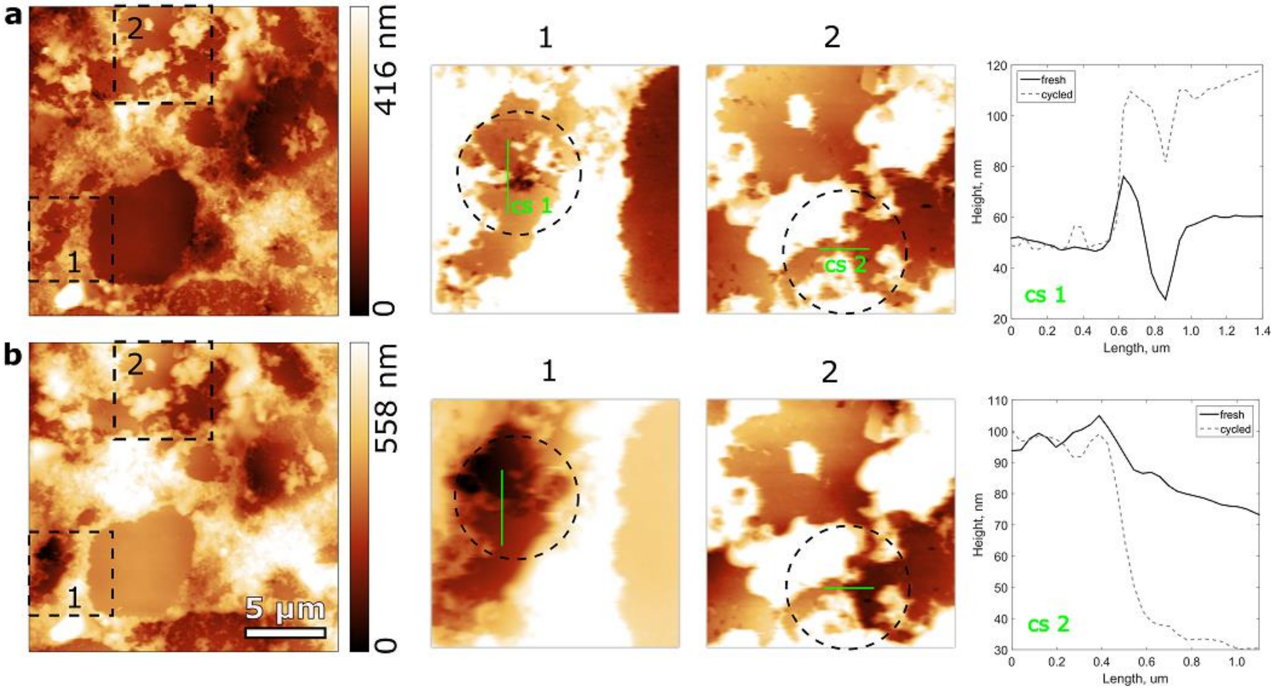

Supplementary Figure 3. AFM topography of NMC 111 before (a) and after (b) cycling. The marked regions are enlarged and cross-sections are shown to illustrate morphological transformations.

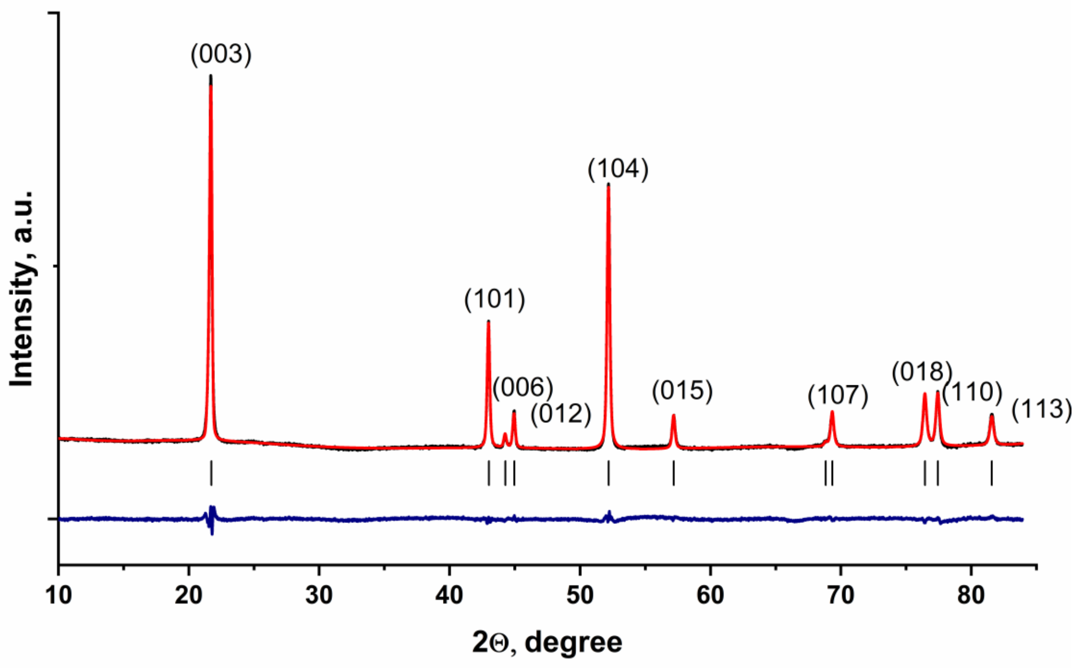

Supplementary figure 4. XRD spectra of $LiNi_{1/3}Co_{1/3}Mn_{1/3}O_2$ powder: a = 2.86003(2), c = 14.2425(2) A, V = 100.8923(2) A^3.

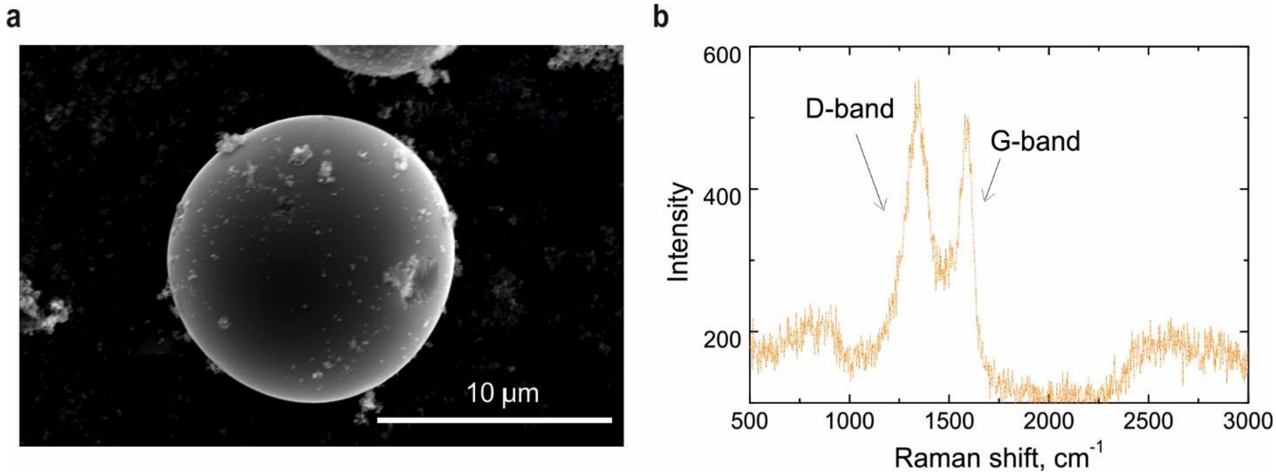

Supplementary figure 5. SEM image (a) and Raman spectrum (b) of the HC powder.

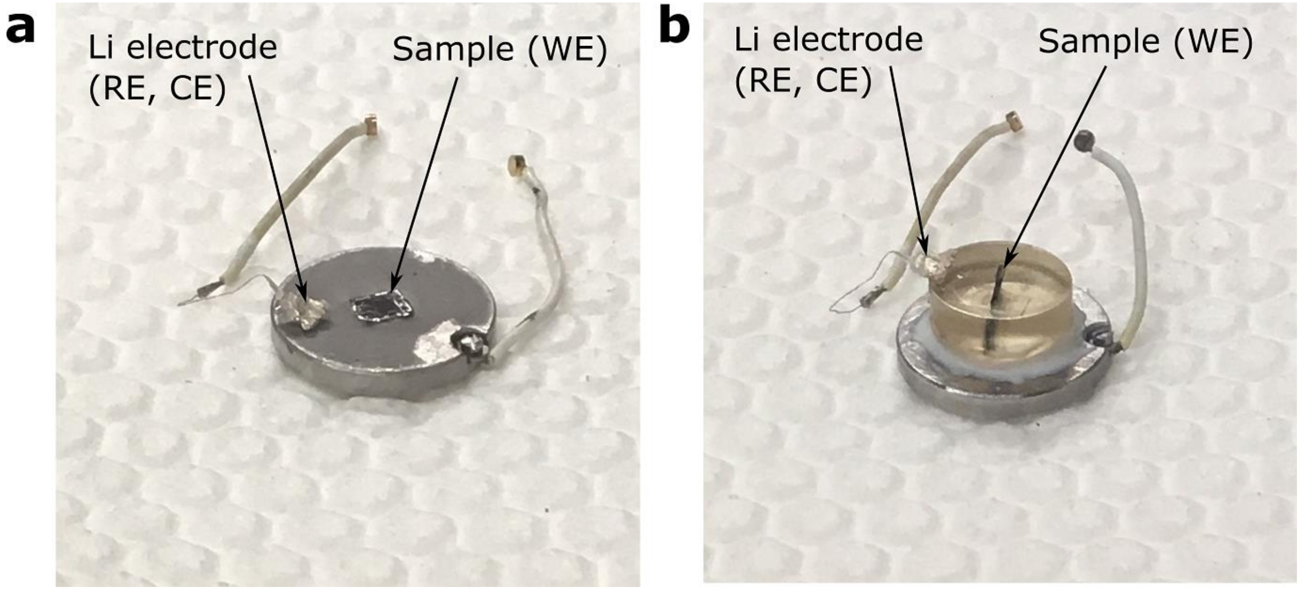

Supplementary figure 6. HOPG (a) and a composite sample (b) mounted on a steel substrate with a contact wire. A piece of Li foil wrapped and pressed around another Cu wire with magnetic connection is shown. The Li is placed on the epoxy that serves both as a sample holder and as a support for Li and electrolyte.

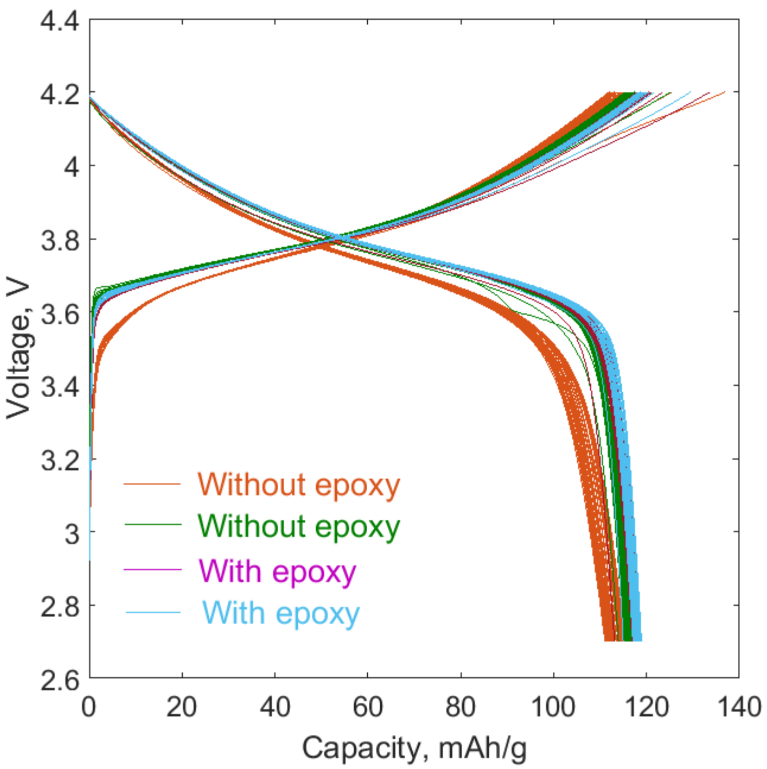

Supplementary figure 7. Comparative cycling of NMC 111/Li half cells with commercial 1M LiPF6 in EC/DMC=50/50 (v/v) electrolyte solution with and without epoxy resin. 0.3C rate, 30 cycles.

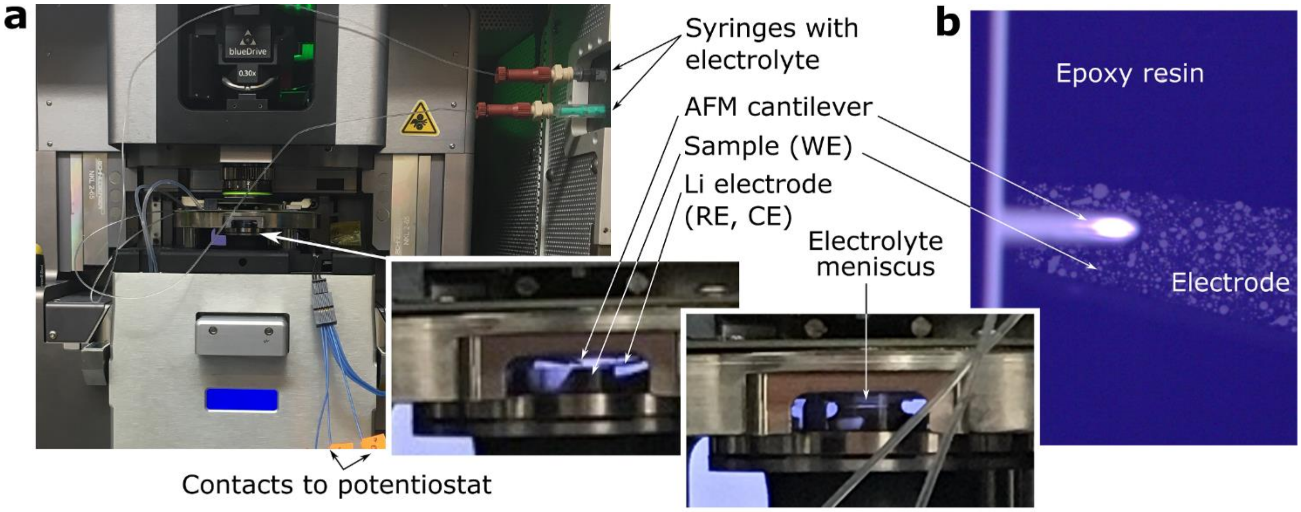

Supplementary figure 8. Experimental setup: general view (a) and optical image of the sample and the AFM cantilever (b). AFM cantilever was mounted in a liquid perfusion holder with a fused silica window. The cantilever was pre-engaged to about 100 um from the sample surface and electrolyte was injected from a syringe through the tubing to the space between the sample and the fused silica window where it formed a meniscus.
